# Supplementary material for: Body Mass Index and Birth Weight Improve Polygenic Risk Score for Type 2 Diabetes
Source: J Pers Med. 2021 Jun 21;11(6):582. doi: 10.3390/jpm11060582 (PMC8233887; doi:10.3390/jpm11060582)
Supplement: Supplementary file 1 [file jpm-11-00582-s001.zip › jpm-1246137-supplementary.pdf]

Body mass index and birth weight improve polygenic risk score for type 2 diabetes

Avigail Moldovan[1#], Yedael Y. Waldman[2#], Nadav Brandes[3] and Michal Linial[1*]

[1] Department of Biological Chemistry, Institute of Life Sciences, The Hebrew University of Jerusalem, Jerusalem, 91904, Israel. [2] NRGene Ltd., Ness-Ziona, Israel. [3] The Rachel and Selim Benin School of Computer Science and Engineering, The Hebrew University of Jerusalem, Jerusalem, 91904, Israel

[#] *These authors contributed equally to this work.* [*] *Corresponding author: michall@mail.huji.ac.il*

Supplementary Figures

S1, S2

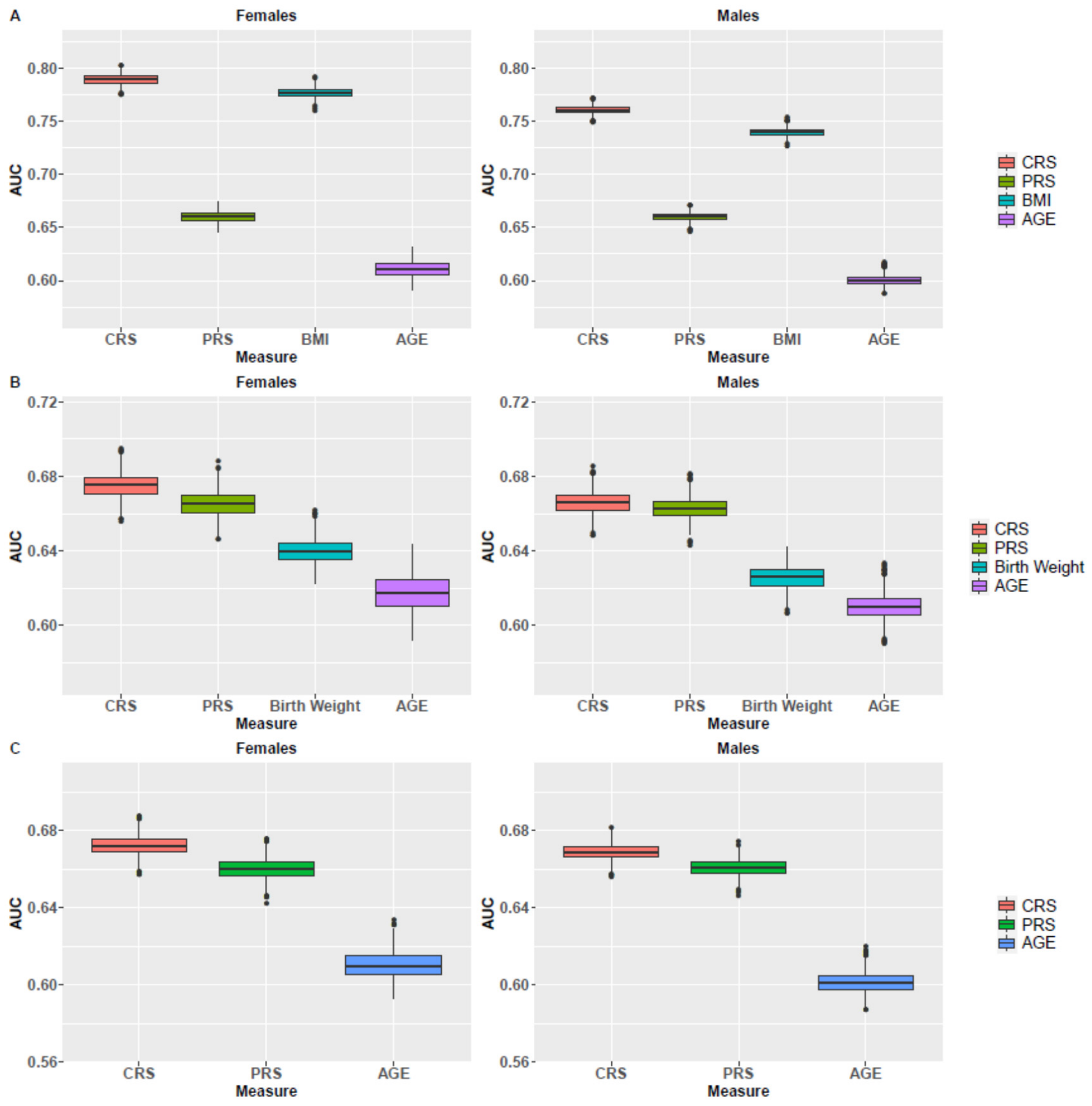

Figure S1. Comparison of AUC values in the test sets for the different measures. In each analysis (BMI, birth weight and comparative body size at age ten), we compared the AUC values of the different measures across 1000 random test sets. Results are shown for the analysis of PRS with (**A**) BMI, (**B**) birth weight and (**C**) comparative body size at age ten. In all cases, the AUC of the combined measure (CRS) achieved significantly higher values as compared to the AUC of all other measures (Wilcoxon signed rank test P-value<10^{-16}).

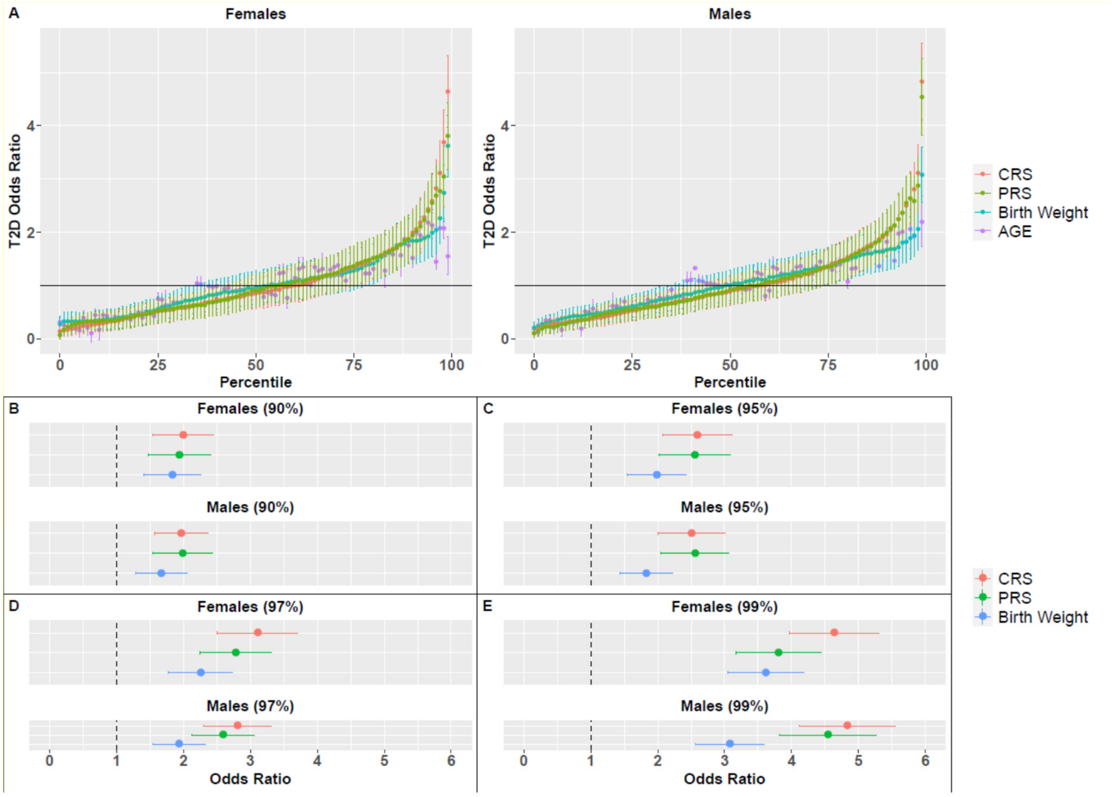

Figure S2. Odds ratio (OR) for T2D, based on birth weight, PRS, CRS or age percentiles. (**A**) OR for all percentiles and all measures for females and males. Vertical lines correspond to the standard deviation of the average OR across 1000 random splits of the dataset. The horizontal line represents a neutral OR of 1. OR values for females and males in specific percentiles are also presented: (**B**) 90th, (**C**) 95th, (**D**) 97th and (**E**) 99th.
